# Combined application of sheep manure and organic fertilizer improves soil quality and microbial community structure and function in alpine mining areas

**DOI:** 10.1128/spectrum.00840-25

**Published:** 2025-08-05

**Authors:** Zhongyang Yu, Xiaoting An, Shengbin Hu, Mingchun Yang, Jianing Li, Changhui Li

**Affiliations:** 1 College of Agriculture and Animal Husbandry, Qinghai University 207475, Xining, China; 2 Veterinary Medicine and Academy of Animal Science, Qinghai University 207475, Xining, China; University of Delhi, Delhi, India

**Keywords:** Alpine mining area, commercial organic fertilizer, sheep manure, soil microorganisms, soil quality

## Abstract

The harsh soil conditions in alpine mining areas severely restrict plant growth, necessitating the urgent development of an optimal fertilization strategy to facilitate soil restoration and ecosystem recovery. Six treatments were incorporated: control with no fertilization (CK), 100% sheep manure (S), 100% commercial organic fertilizer (F), 60% sheep manure + 40% commercial organic fertilizer (M1), 50% sheep manure + 50% commercial organic fertilizer (M2), and 40% sheep manure + 60% commercial organic fertilizer (M3). The treatments’ impacts on soil properties, microbial composition, functionality, and their interactions were systematically analyzed. The combined application of sheep manure and commercial organic fertilizer significantly enhanced soil organic matter (SOM) and available nutrients. The findings revealed that the M1 treatment showed the greatest improvement, with total nitrogen (TN), total phosphorus (TP), SOM, available nitrogen (AN), and available phosphorus (AP) increasing by 211.07%, 136.27%, 388.18%, 564.97%, and 282.53%, respectively, in comparison to the CK treatment. Nutrient addition significantly altered the structure of soil microbial communities and the abundance of functional microorganisms. But it had no significant effect on the Shannon and Simpson indices of the soil fungal community. Key factors such as TP, SOM, TN, AP, and AN significantly influenced bacterial distribution, while TP, AN, and AP played a crucial role in fungal distribution. Bacterial diversity and fungal functionality were mainly regulated by TP, while bacterial functionality was primarily controlled by pH and SOM. In conclusion, the M1 treatment appears to be a viable strategy for promoting soil restoration in alpine mining areas.

**IMPORTANCE:**

Ecological restoration in mining areas is a global challenge. We systematically investigated the effects of sheep manure, commercial organic fertilizer, and their combined application on soil physicochemical properties, microbial community structure, and functions in the Muli mining area of the Qinghai-Tibetan Plateau. Results showed that the combined application significantly improved soil quality, with the optimal ratio being 60% sheep manure and 40% commercial organic fertilizer. Furthermore, the study revealed the mechanisms by which nutrient addition enhances soil quality by analyzing the relationships between soil properties and microbial communities under different treatments. These findings provide valuable insights for restoring ecosystem functions in alpine mining areas of the Qinghai-Tibetan Plateau and promoting sustainable grassland agriculture.

## INTRODUCTION

The Muli mining area, situated in the northeastern part of the Tibetan Plateau, is currently experiencing significant soil degradation issues (1, 2). Prolonged coal mining activities have disrupted the natural soil structure, leading to soil erosion and a decline in fertility (3). The region’s distinct geological characteristics have been adversely affected, with the permafrost layers being damaged by mining operations, which in turn impact soil moisture retention and stability (1, 4). Moreover, the destruction of surface vegetation due to coal extraction has eliminated its stabilizing and protective role, thereby worsening soil erosion (3, 5). Additionally, changes in hydrology, pollution, and climate change resulting from excessive mining have further exacerbated the degradation of the soil environment (6, 7). These compounded factors have led to a notable decline in soil quality, posing a considerable threat to the ecological stability of the Tibetan Plateau (2). Therefore, it is imperative to investigate efficient strategies for improving soil quality in the Muli mining area.

Establishing artificial grasslands serves as an effective strategy for the restoration of soil quality in alpine mining regions, though it must be accompanied by suitable management practices (8, 9). The prolonged human exploitation and disturbance of these areas have led to substantial soil degradation, including impoverishment, structural deterioration, and salinization (10). These fundamental deficiencies pose significant challenges for rapid soil improvement through artificial grasslands, thereby requiring extended periods of natural recovery coupled with human intervention (11). During the initial phase of artificial grassland establishment, supplementary nutrition, such as fertilization, may be essential (12). A lack of adequate nutritional supplementation can hinder plant growth, ultimately reducing the efficacy of soil remediation. Consequently, when constructing artificial grasslands in alpine mining areas, it is crucial to first select appropriate methods for modifying the soil tillage layer structure, followed by the judicious application of organic fertilizers during seeding (2). Sheep manure, which serves as a structural material capable of carrying soil particles while being rich in nutrients (13, 14), has found widespread use in soil pollution control. However, due to its relatively low nutrient content, sheep manure application generally requires supplementation with commercial organic fertilizers (15, 16). The combined application of manure and organic fertilizers offers complementary nutrients, thereby enhancing the soil nutrient balance (17, 18). This combination also facilitates soil aggregate formation, thereby enhancing soil aeration and permeability (17). However, varying types and proportions of manure and organic fertilizer combinations lead to different impacts on soil physicochemical properties (19, 20). Research has suggested that the combined use of pig and chicken manure increases soil organic matter (SOM) and nutrient content (21, 22), while the combination of cattle manure with straw incorporation enhances soil structure and improves drought resistance (23, 24). In conclusion, the combined application of manure and commercial organic fertilizers markedly improves soil physicochemical properties. However, for effective remediation of soil degradation in alpine mining areas, the appropriate selection of manure type and its ratio with commercial organic fertilizers is essential to achieve optimal soil improvement.

Soil microorganisms are pivotal in soil restoration, engaging in intricate biochemical processes that enhance soil fertility, structure, ecological equilibrium, and environmental preservation (25–27). Studies suggest that the simultaneous use of manure and commercial organic fertilizers markedly boosts soil microbial diversity (28, 29). High-throughput sequencing-based analyses demonstrate that the application of these combined treatments leads to a richer and more diverse microbial community structure (30, 31). Furthermore, this dual application is known to modify the composition of the microbial community, fostering the proliferation of beneficial microorganisms like bacteria, fungi, and actinomycetes (32). These microorganisms serve essential functions in soil, further enhancing its fertility. The deployment of manure and commercial organic fertilizers can also affect the abundance and activity of functional genes within the soil microbial population (32). For instance, the introduction of manure increases the prevalence of genes associated with phosphorus cycling, thus promoting microbial mineralization of organic phosphorus and the solubilization of inorganic phosphorus (33, 34). In conclusion, the integrated use of manure and commercial organic fertilizers exerts considerable influence on the structure and function of soil microbial communities. Nevertheless, to optimize the microbial community structure and maximize its functionality in soil restoration efforts, the ideal application ratio must be identified.

In this study, commercial organic fertilizer and sheep manure sourced from the vicinity of the Muli mining region were chosen as test fertilizers. A range of treatments was established, including both individual applications and various proportion combinations (control [CK], 100% sheep manure [S], 100% commercial organic fertilizer [F], 60% sheep manure + 40% commercial organic fertilizer [M1], 50% sheep manure + 50% commercial organic fertilizer [M2], and 40% sheep manure + 60% commercial organic fertilizer [M3]). The physicochemical properties of the soil, along with microbial community structure and functionality, were measured and analyzed in the second year. The objectives of this investigation were (i) to assess the impacts of different nutrient addition treatments on the soil microbial community structure and function in alpine mining areas; (ii) to identify the key factors driving the improvement of soil microbial diversity and function through nutrient addition in these areas; and (iii) to establish the most effective nutrient addition strategy for soil restoration in alpine mining environments.

## MATERIALS AND METHODS

### Experimental site description

The research site is situated within the Juhugeng mining region of the Muli coalfield, situated in the northeastern Qinghai-Tibet Plateau within the Haixi Mongolian and Tibetan Autonomous Prefecture, Tianjun County, Qinghai Province (99.05°–99.27°E, 38.05°–38.27°N), with an average altitude of approximately 3,800–4,200 m, predominantly characterized by high-altitude periglacial landforms. The natural vegetation types in the mining area are classified as alpine marshes and alpine meadows, displaying distinct physical characteristics of alpine regions, with simple plant community structures, sparse vegetation, and weak resistance to human activities. The Juhugeng mining area features a classic plateau continental climate, showcasing chilly temperatures and notable fluctuations in daily temperature. The rainy season occurs from June to August, while snowfall predominates from November to May of the following year. The yearly mean temperature is −4.2°C, with an average annual rainfall of around 477.1 mm, and an average annual evaporation of 1,049.9 mm. With long winters and no summers, the region falls within the Qilian Mountains’ high-altitude permafrost zones, where permafrost is extensively developed. The thickness of the perennially frozen ground ranges from 40 to 160 m, with an average thickness of 120 m, and the permafrost layer starts at depths of 0.95 to 5.50 m. Before vegetation restoration on the mining slag heap, the physicochemical properties of the topsoil were as follows: total nitrogen 1.17 g·kg^−1^, total phosphorus 0.91 g·kg^−1^, available nitrogen 18.00 g·kg^−1^, available phosphorus 6.70 mg·kg^−1^, organic matter 93.33 g·kg^−1^, and pH 8.46.

### Determination of research subjects

On 29 June 2022, a coal storage site was chosen for the experimental plot layout. The soil was tilled to a depth of about 30 cm with a ripper, larger rocks within the plot were removed, sheep slab manure and granular organic fertilizers were mixed with the soil using diggers, disc harrows, and manual methods. The soil tillage layer was approximately 30 cm. Four treatments were established: CK (no fertilization), S (100% sheep manure), F (100% commercial organic fertilizer), M_1_ (60% sheep manure + 40% commercial organic fertilizer), M_2_ (50% sheep manure + 50% commercial organic fertilizer), and M_3_ (40% sheep manure + 60% commercial organic fertilizer), with specific application rates detailed in Table 1. A randomized block design was implemented with each treatment replicated three times, totaling 12 plots, each with an area of 6 m × 5 m. This study selected grass species native to the Qinghai-Tibet Plateau that are well-suited to the local environment: *Poa pratensis cv. Qinghai*, *Poa crymophila cv. Qinghai*, *Festuca sinensis cv. Qinghai*, and *Elymus sibiricus cv. Tongde*, with a total sowing rate of 22.5 g·m^−2^. Equal amounts of forage-specific fertilizer (total nutrients ≥35%, N 18%, P_2_O_5_ 12%, K_2_O_5_ %) and seeds were thoroughly mixed in sealed pots, and then uniformly spread on the cultivated layer that had been manually furrowed beforehand, at a sowing depth of 1 cm. The seeds were covered with soil and then trodden down with feet and covered with non-woven fabric to insulate and promote germination.

**TABLE 1:** Different fertilization treatments, fertilizer ratios, and application rates^*a*^

Treatment	Organic fertilizer		Sheep board manure	
	Rate/kg·m^−2^	Proportion/%	Rate/m^−3^·m^−2^	Proportion/%
CK	0	0	0	0
S	0	0	0.05	100
F	2.5	100	0	0
M1	1	40	0.03	60
M2	1.25	50	0.025	50
M3	1.5	60	0.02	40

^*a*^ CK, no fertilization; S, 100% sheep manure; F, 100% commercial organic fertilizer; M1, 60% sheep manure + 40% commercial organic fertilizer; M2, 50% sheep manure + 50% commercial organic fertilizer; M3, 40% sheep manure + 60% commercial organic fertilizer.

### Soil sampling and determination

On 30 July 2023, a five-point sampling technique was employed to gather 0–10 cm soil samples from each plot using a 3.5 cm diameter soil auger. The soil specimens obtained from the five points were combined and separated into two portions: one portion was sifted through a 1 mm screen for analysis of soil nutrient content, while the other portion was placed in sterile 50 mL microfuge flasks and stored at −80°C for ultra-high sequencing of microbes.

Soil pH was determined using a pH meter (Sartorius PB-10, Germany) at a 1:2.5 soil to water ratio (35); SOM was determined using the potassium dichromate oxidation method (36). Total phosphorus (TP) was measured by colorimetry after wet digestion with H_2_SO_4_ and H_2_O_2_ (UV2800A UV-Vis Spectrophotometer, UNIC Inc., China) (37). Available nitrogen (AN) was determined using the alkali diffusion method (38). Total nitrogen (TN) content was measured using an elemental analyzer (FLASH SMART CHNS/O, Germany). Available phosphorus (AP) was determined using the molybdenum blue method (39).

### DNA extraction, PCR amplification, and high-throughput sequencing

Use the DNeasy Power-Soil Kit (QIAGEN) to extract total DNA of soil microorganisms. The *in vitro* extraction of DNA from microbial genomic sources was conducted using the E.Z.N.A. Soil samples were utilized as the source material. The 1% agarose gel electrophoresis and NanoDrop2000 TM spectrophotometer (Thermo Scientific, USA) were employed to assess the quality and concentration of the DNA samples. Following this, the samples were stored at −80°C *in vivo* until they were subjected to further analysis. The V3-V4 hypervariable regions of the bacterial 16S rRNA gene were amplified with primers 338F (5′-ACTCCTACGGGAGGCAGCAG-3′) and 806R (5′-GGACTACHVGGGTWTCTAAT-3′) using a T100 Thermal Cycler PCR (BIO-RAD, USA) (40). Fungi were selected to amplify the ITS1 region fragments using ITS 5-1737F and ITS 2-2043R primers. PCR products were extracted from a 2% agarose gel, purified using a PCR Purification Kit (YuHua, Shanghai, China) as per the manufacturer’s instructions, and quantified using a Qubit 4.0 (Thermo Fisher Scientific, USA).

### Sequencing process and bioinformatics approaches

The FASTQ files were demultiplexed using in-house Perl scripts and then underwent quality filtering with fastp version 0.19.6 (41). The data were merged using FLASH version 1.2.7 (42) based on the specified criteria. The *de novo*-generated sequences were subsequently clustered into operational taxonomic units (OTUs) using UPARSE 7.1 (43) at a 97% identity threshold. The most prevalent sequence within each OTU was identified as a representative sequence. In order to mitigate the impact of sequencing depth on α and β diversity calculi, the number of 16S rRNA gene sequences per sample was rarefied to 20,000, thereby achieving an average Good’s coverage of 99.09%. The *de novo* synthesized 250-nucleotide amplicons were pooled in equimolar ratios and sequenced on the Illumina PE300/PE250 platform (Illumina, San Diego, USA) in accordance with the standard protocols provided by Majorbio Bio-Pharm Technology Co., Ltd. (Shanghai, China).

### Statistical analysis

An analysis of variance was conducted using SPSS 27.0 to assess variations in aboveground biomass, soil properties, and microbiological properties across different treatments. Principal coordinate analysis (PCoA) was utilized to explore the *in situ* microbial community structures of samples, employing the Bray-Curtis distance algorithm. Linear discriminant analysis Effect Size (LEfSe) was utilized to detect bacterial and fungal taxa with substantial abundance variances at the phylum and genus levels across diverse groups. The analysis criteria included a linear discriminant analysis (LDA) score greater than 2 and a significance level of *P* < 0.05. The analysis was conducted by accessing the following link: http://huttenhower.sph.harvard.edu/LEfSe (44). Utilizing FAPROTAX (http://www.loucalab.com/archive/FAPROTAX/) and FUNGuild (https://github.com/UMNFuN/FUNGuild), the functions of bacterial and fungal communities were analyzed and predicted. Redundancy analysis (RDA) was conducted to evaluate the influence of soil physicochemical parameters on the structures of soil microbial communities. Heatmaps depicting the correlation between soil physicochemical properties and microbial community traits were generated utilizing Pearson correlation coefficients. The visualization was created using the online pipeline available at https://www.omicstudio.cn/tool/109. Structural equation modeling (SEM) was performed using the R software version 4.0.2. The overall fit of the piecewise SEM was evaluated using Shipley’s test of d-separation and Fisher’s C statistic to test whether any paths were missing from the model in the r package piecewise SEM. We reported the standardized coefficient for each path from each component model. Box line plots of microbial community diversity and chord plots of microbial community composition were plotted using Origin 2022 software.

## RESULTS

### Effects of nutrient addition on soil physicochemical properties

Nutrient addition was found to markedly influence the physicochemical properties of the soil (Table 2, *P* < 0.05). The pH level of the soil under the M2 treatment was notably elevated versus that of the other treatments (Table 2). In comparison to the control group, treatments involving single applications generally led to a decrease in soil pH, whereas those with mixed applications exhibited an increase in soil pH. The concentrations of SOM and available nutrients under the mixed application treatments were considerably higher than those observed in other treatments. Specifically, the M1 treatment was responsible for a significant increase in the levels of total nitrogen (TN), total phosphorus (TP), organic matter (OM), available nitrogen (AN), and available phosphorus (AP) compared to the other treatments. When compared to the CK, M1 treatment resulted in an increase of 211.07% in TN, 136.27% in TP, 388.18% in OM, 564.97% in AN, and 282.53% in AP (Table 2).

**TABLE 2:** Soil physical and chemical characteristics following various treatments^*a*^

Treatments	pH	TN (g·kg^−1^)	TP (g·kg^−1^)	SOM (g·kg^−1^)	AN (mg·kg^−1^)	AP (mg·kg^−1^)
CK	7.74 ± 0.02bc	3.07 ± 0.12e	1.02 ± 0.04e	35.62 ± 0.45f	13.39 ± 0.47f	33.15 ± 2.36f
S	7.55 ± 0.08c	6.94 ± 0.42b	1.81 ± 0.04b	109.42 ± 3.29d	44.52 ± 1.03d	76.35 ± 6.49d
F	7.50 ± 0.01c	4.43 ± 0.11d	1.23 ± 0.03d	90.09 ± 0.98e	20.68 ± 1.49e	57.30 ± 3.89e
M1	7.75 ± 0.05bc	9.55 ± 0.24a	2.41 ± 0.10a	170.89 ± 4.86a	89.01 ± 1.64a	126.81 ± 2.96a
M2	8.06 ± 0.14a	5.97 ± 0.45c	1.94 ± 0.06b	137.50 ± 2.56b	63.87 ± 0.96b	104.94 ± 2.93b
M3	7.90 ± 0.11ab	5.78 ± 0.25c	1.63 ± 0.04c	125.47 ± 3.27c	56.66 ± 3.05c	92.03 ± 3.11c

^*a*^ Statistically significant differences between treatments are indicated by different lowercase letters (*P* < 0.05). CK, no fertilization; S, 100% sheep manure; F, 100% commercial organic fertilizer; M1, 60% sheep manure + 40% commercial organic fertilizer; M2, 50% sheep manure + 50% commercial organic fertilizer; M3, 40% sheep manure + 60% commercial organic fertilizer; TN, total nitrogen; TP, total phosphorus; SOM, soil organic matter; AN, available nitrogen; AP, available phosphorus.

### Effects of nutrient addition on soil microbial communities

#### Effects on soil microbial diversity

**Fig 1:**
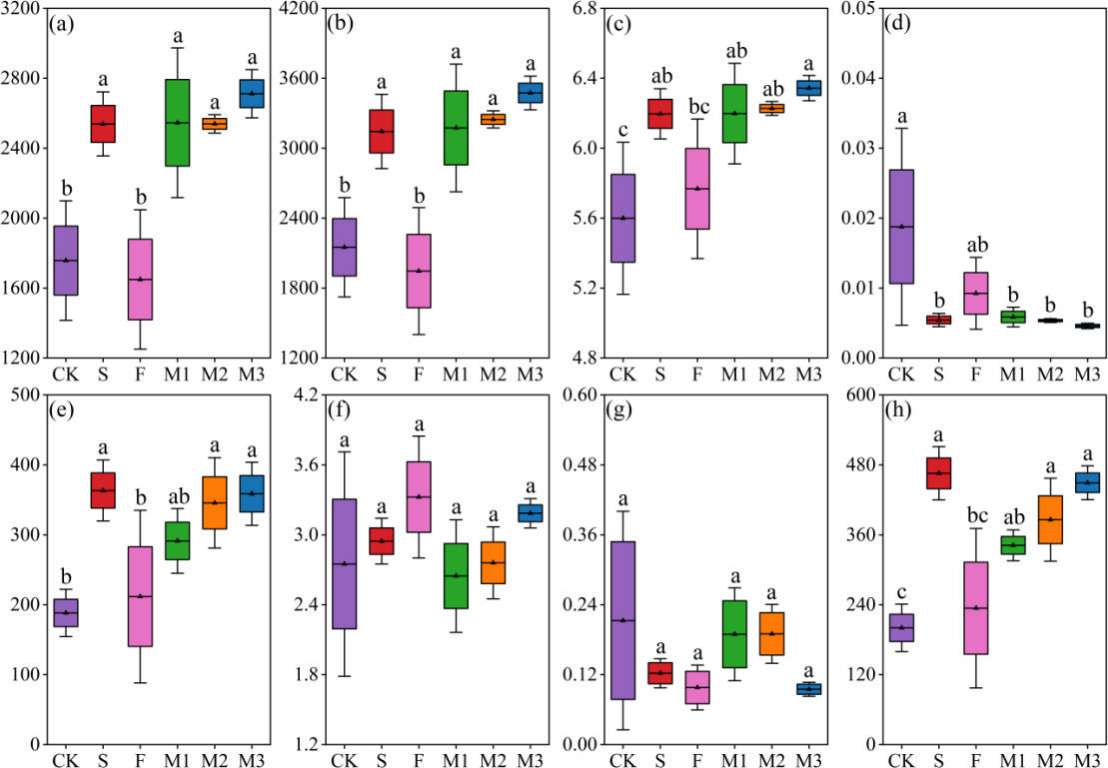
Alpha diversity of soil bacterial and fungal communities under different treatments. Note: figures (a) to (d) show the OTUs index, Chao’s index, Shannon’s index, and Simpson’s index of the soil bacterial community, respectively. Figures (e) to (h) showed the OTUs index, Chao’s index, Shannon’s index, and Simpson’s index of soil fungal communities, respectively. CK, no fertilization; S, 100% sheep manure; F, 100% commercial organic fertilizer; M1, 60% sheep manure + 40% commercial organic fertilizer; M2, 50% sheep manure + 50% commercial organic fertilizer; M3, 40% sheep manure + 60% commercial organic fertilizer.

Nutrient addition was shown to markedly impact the Alpha diversity of soil microbial communities (Fig. 1, *P* < 0.05). In comparison to CK, the treatments S, M1, M2, and M3 were associated with substantial increases in the OTU index, Chao index, and Shannon index of soil bacterial communities (Fig. 1a through c), accompanied by a significant reduction in the Simpson index (Fig. 1d). Significant increases in the OTU and Chao indices of soil fungal communities were observed in treatments S, M2, and M3 (Fig. 1e and f). Nevertheless, the Shannon and Simpson indices of soil fungal communities remained largely unaffected by the various nutrient addition treatments (Fig. 1g and h).

**Fig 2:**
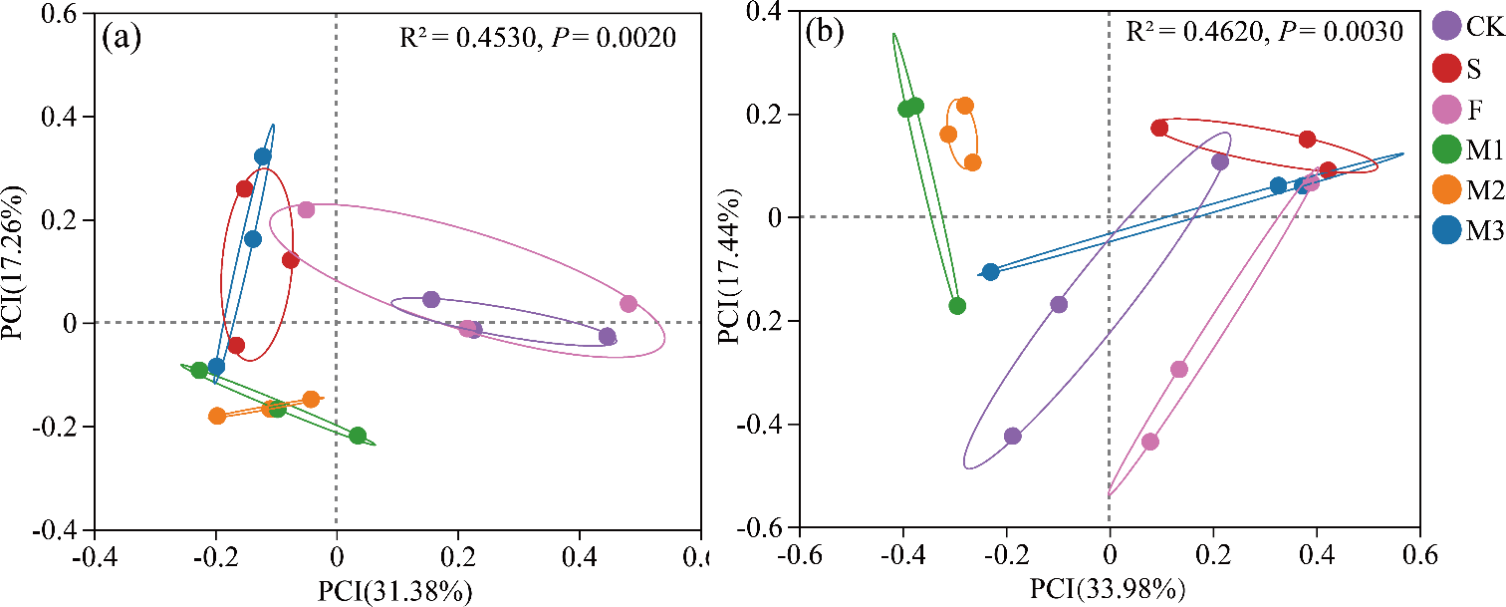
PCoA analysis based on Bray-Curtis distance of soil bacterial communities (a) and fungal communities (b) under different treatments.

Principal coordinate analysis demonstrated that nutrient addition notably affected the Beta diversity of soil microbial communities (Fig. 2, *P* < 0.05). The variance of the two principal components collectively accounted for 48.64% of the bacterial community, with notable differences observed between groups (*R* = 0.3673, *P* = 0.0020, Fig. 2a). Similarly, the two principal components explained 51.42% of the fungal community variance, with significant group differences identified (*R* = 0.4620, *P* = 0.0030, Fig. 2b). Bacterial and fungal communities under the various treatments were distinctly clustered into six separate groups, corresponding to the six nutrient addition treatments.

#### Effects on soil microbial community structure

**Fig 3:**
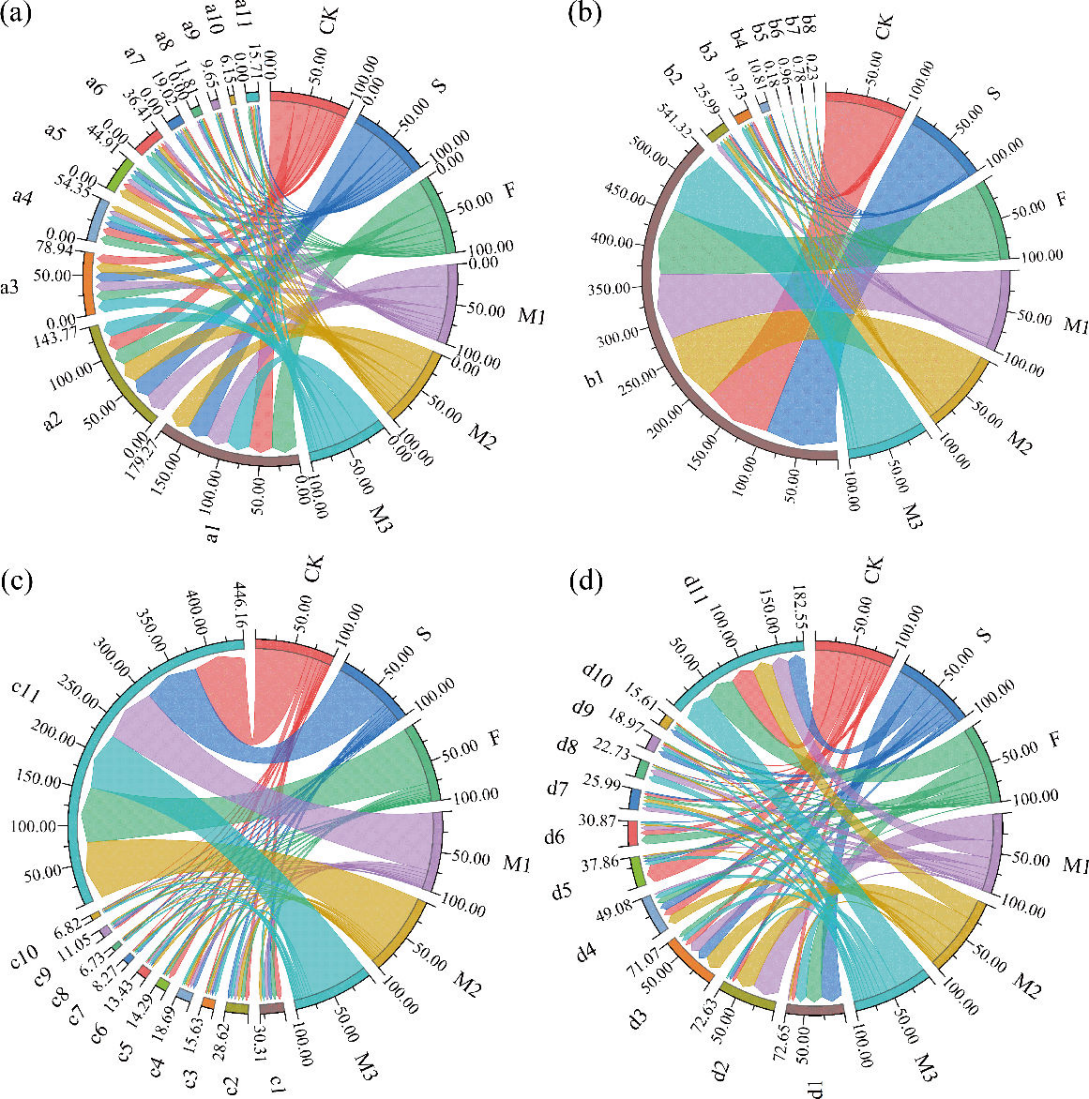
Relative abundance of soil microbial communities at the phylum and genus levels across various treatments. Note: (a) and (c) indicate the relative abundance of bacterial communities at the phylum and genus levels, while (b) and (d) represent the relative abundance of fungal communities at the phylum and genus levels. a1–a11 represent Proteobacteria, Actinobacteriota, Chloroflexi, Bacteroidota, Firmicutes, Acidobacteriota, Myxococcota, Gemmatimonadota, Patescibacteria, Verrucomicrobiota, and others, respectively; b1–b8 represent Ascomycota, Unclassified_Fungi, Basidiomycota, Chytridiomycota, Olpidiomycota, Mortierellomycota, Monoblepharomycota, and others, respectively; c1–c11 represent *Sphingomonas, Norank_JG30-KF-CM45, Norank_A4b, Nocardioides, Pseudarthrobacter, Devosia, Unclassified_Microbacteriaceae, Norank_Vicinamibacterales, Altererythrobacter, OLB13* and others, respectively; d1–d11 represent *Thelebolus, Unclassified__Lasiosphaeriaceae, Schizothecium, Preussia, Unclassified__Sordariales, Gibberella, Unclassified__Fungi, Kernia, Unclassified_f__Sporormiaceae, Cephalotrichum,* and others, respectively.

The composition of the bacterial community was examined under various nutrient-addition treatments. Ten dominant bacterial phyla, including Proteobacteria, Actinobacteria, Chloroflexi, and Bacteroidota, were identified (Fig. 3a), along with seven dominant fungal phyla, including Ascomycota, Basidiomycota, and Chytridiomycota (Fig. 3b). Furthermore, 10 dominant fungal genera, including *Thelebolus*, *Schizothecium*, and *Preussia* (Fig. 3d), and 10 dominant bacterial genera, including *Sphingomonas*, *Norank_JG30-KF-CM45*, and *Norank_A4b* (Fig. 3c), were noted. The relative abundance of Acidobacteriota, Monoblepharomycota, *Thelebolus*, *Preussia*, and Cephalotrichum was much lower with M1 treatment than under the other treatments, but the relative abundance of Gemmatimonadota and Mortierellomycota was significantly higher. The relative abundance of *Sphingomonas*, *Norank_JG30-KF-CM45*, *Norank_A4b, Nocardioides*, *Pseudarthrobacter*, and *Altererythrobacter* was greatly decreased by nutrient addition, whereas the relative abundance of *Norank_A4b* and *OLB13* was significantly enhanced.

**Fig 4:**
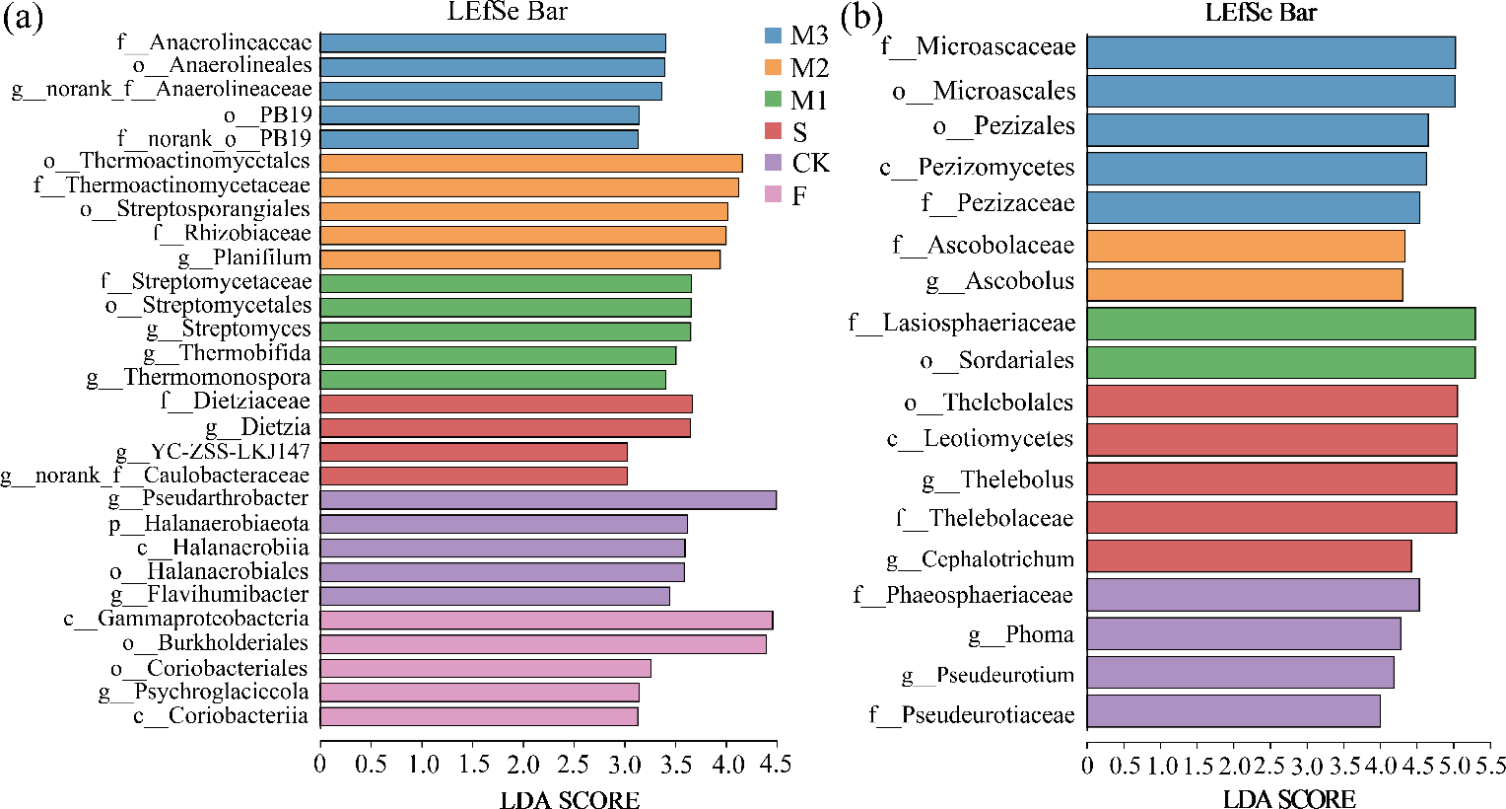
LDA score histograms were calculated for taxa with different abundances in soil bacterial communities (a) and fungal communities (b) under different treatments. c, Class; o, Order; f, Family; g, Genus.

Biomarkers in microbial communities under various nutrient addition treatments were found using LEfSe analysis (Fig. 4). LDA analysis identified 29 biomarkers with considerable biostatistical significance in the bacterial community (CK = 5, S = 4, F = 5, M1 = 5, M2 = 5, M3 = 5) (Fig. 4a). Pseudarthrobacter (g), Dietziaceae (f), Gammaproteobacteria (c), and Streptomycetaceae (f) were the biomarkers with the highest scores at the categorical level for CK, S, F, M1, M2, and M3 treatments, followed by Thermoactinomycetales (o) and Anaerolineaceae (f) (Fig. 4a). Sixteen biomarkers with considerable biostatistical significance were identified in the fungal community by LDA analysis (CK = 4, S = 5, F = 0, M1 = 2, M2 = 2, M3 = 5) (Fig. 4b). For the CK, S, M1, M2, and M3 treatments, the biomarkers that scored the highest at the taxonomic level were Phaeosphaeriaceae (f), Thelebolales (o), Lasiosphaeriaceae (f), Ascobolaceae (f), and Microascaceae (f), in that order (Fig. 4b).

#### Effects on soil microbial functions

**Fig 5:**
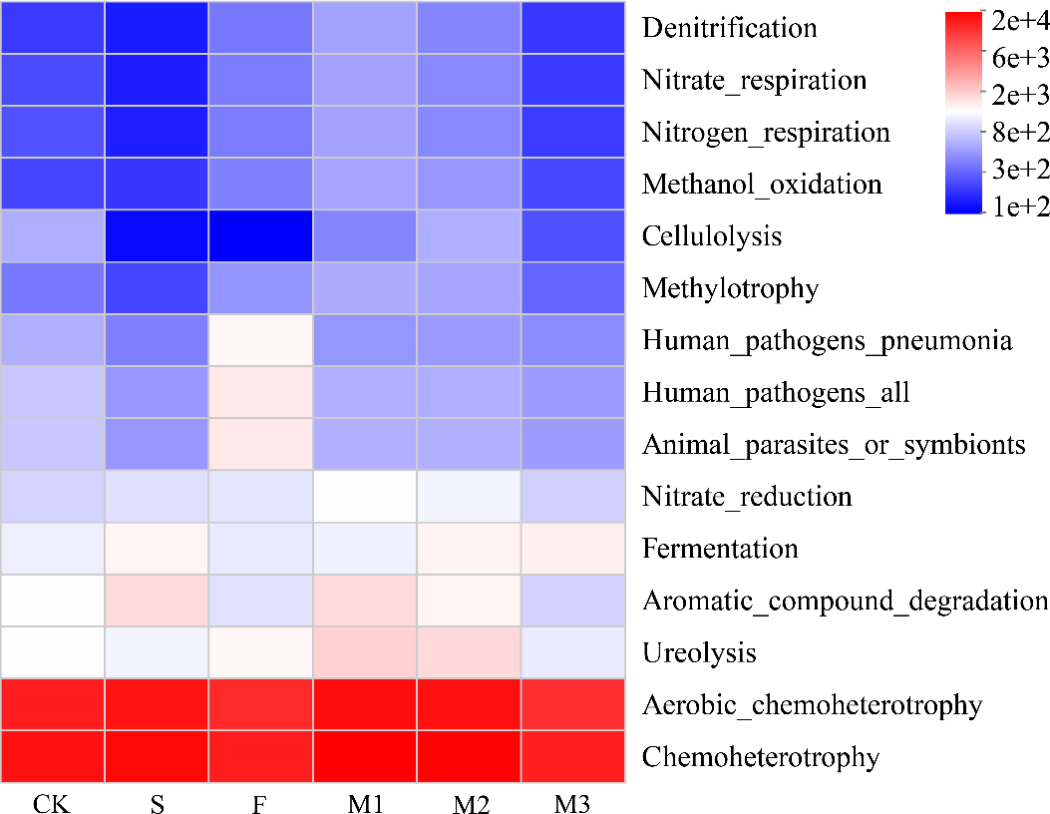
Prediction of soil bacterial function under different treatments.

**Fig 6:**
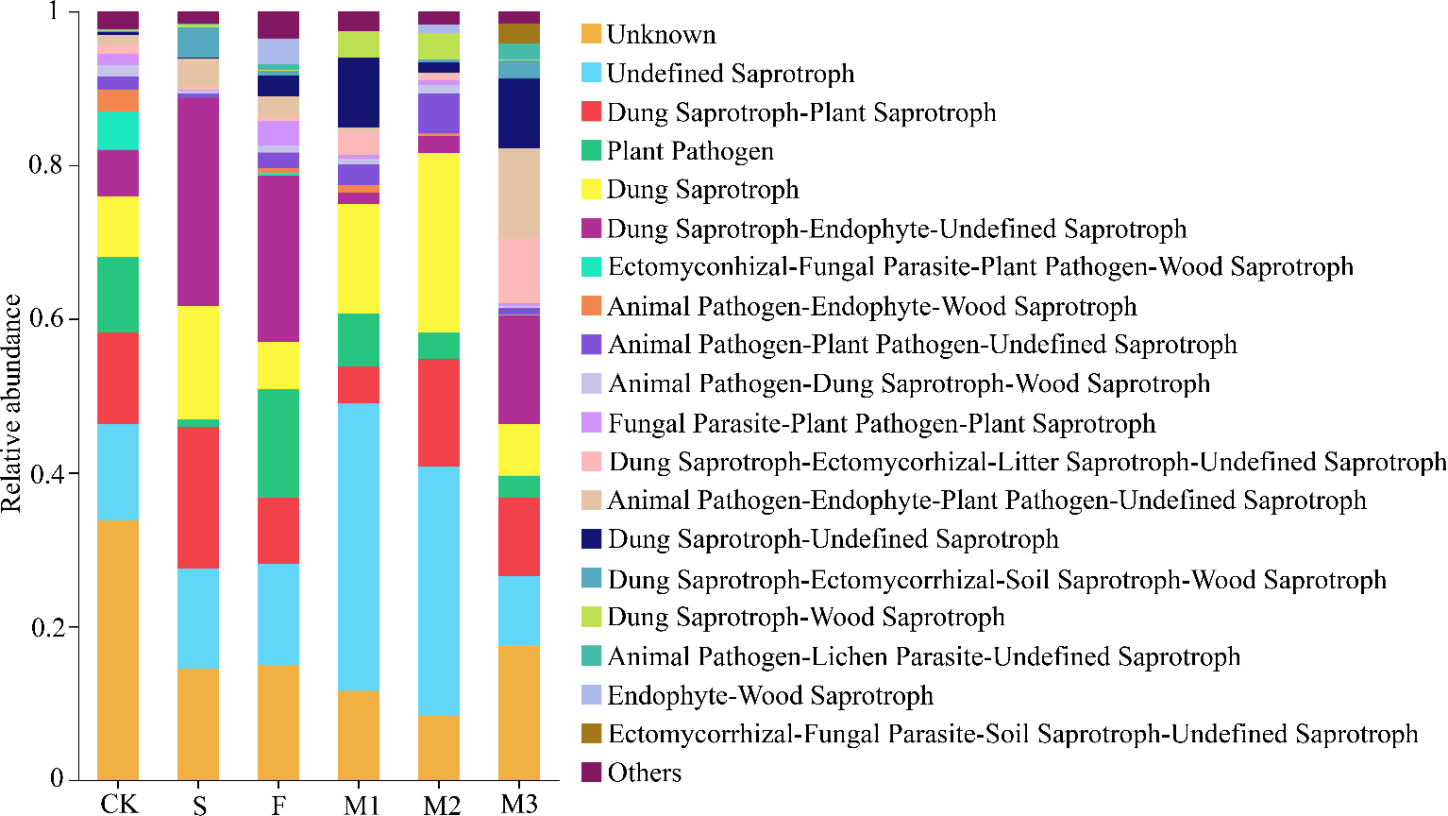
Prediction of soil fungal function under different treatments.

There was a considerable impact of various fertilizer inputs on the relative abundance of soil bacterial and fungal functional microorganisms (Fig. 5 and 6). All the soil samples had bacterial functions including Chemoheterotrophy, Aerobic_chemoheterotrophy, and Ureolysis (Fig. 5), as well as fungal functions like Dung Saprotroph and Plant Pathogen (Fig. 6). The relative abundance of Aromatic_Compound_Degradation was considerably elevated by the S treatment, while the relative abundance of Animal_parasites_or_symbionts and Human_Pathogens_All were significantly elevated by the F treatment. The M1 treatment dramatically decreased the relative abundance of Dung Saprotroph-Plant Saprotroph while greatly increasing Aromatic_compound_degradation, Ureolysis, and Undefined Saprotroph.

### Relationships between soil physical and chemical properties and microbial communities

**Fig 7:**
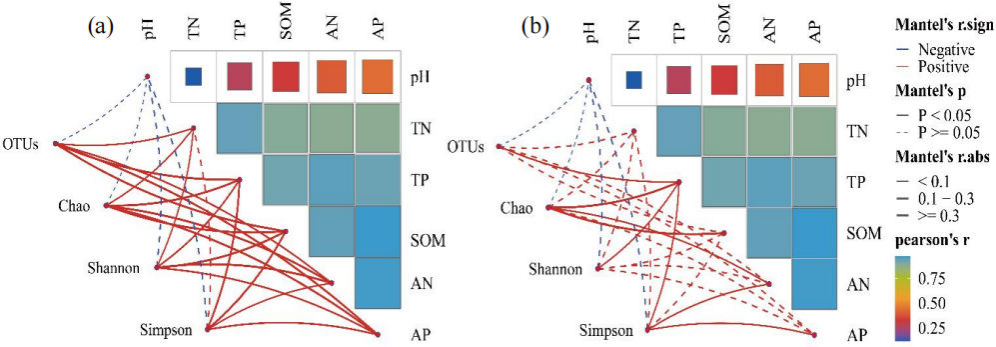
Mantel’s correlations analysis between soil physicochemical properties and bacterial (a) and fungal (b) diversity, respectively. Solid lines indicate significant correlations and dashed lines indicate insignificant correlations. Red lines indicate positive correlations and blue lines indicate negative correlations. Thick and thin lines indicate higher or lower corresponding correlations, respectively.

Heatmap analysis, based on Pearson correlation, revealed that soil pH did not exert a significant influence on bacterial and fungal diversity (Fig. 7, *P* > 0.05). Soil chemical properties, such as TN, TP, OM, AP, and AN, were markedly and positively correlated with bacterial diversity (Fig. 7a, *P* < 0.05). Fungal diversity displayed a significant positive link to TP content in the soil (Fig. 7b, *P* < 0.05).

**Fig 8:**
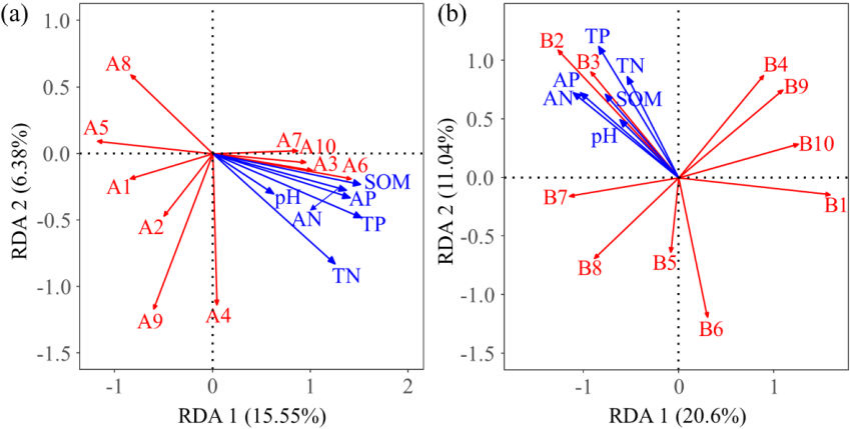
Redundancy analysis of dominant bacterial genera (a) and dominant fungal genera (b) with soil physicochemical properties. In the figure, A1–A10 represent *Sphingomonas*, *Norank_JG30-KF-CM45*, *Norank_A4b*, *Nocardioides*, *Pseudarthrobacter*, *Devosia*, *Unclassified_Microbacteriaceae*, *Norank_Vicinamibacterales*, *Altererythrobacter*, and *OLB13*, respectively; B1–B10 represent *Thelebolus*, *Unclassified__Lasiosphaeriaceae*, *Schizothecium*, *Preussia*, *Unclassified__Sordariales*, *Gibberella*, *Unclassified__Fungi*, *Kernia*, *Unclassified_f__Sporormiaceae*, and *Cephalotrichum*, respectively.

RDA was conducted to examine the relationship between soil physicochemical properties and the dominant bacterial and fungal genera (Fig. 8). In the RDA of soil physicochemical properties and bacterial communities, the two ordination axes collectively accounted for 21.93% of the variation within the community (Fig. 8a). The findings indicated that TP (r² = 0.4446, *P* = 0.013), SOM (r² = 0.4037, *P* = 0.027), TN (r² = 0.3776, *P* = 0.032), AP (r² = 0.3617, *P* = 0.035), and AN (r² = 0.3381, *P* = 0.048) were the primary soil physicochemical factors shaping bacterial community distribution. Similarly, in the RDA involving soil physicochemical properties and fungal communities, 31.64% of the variation in community composition was explained by the two ordination axes (Fig. 8b). The results demonstrated that TP (r² = 0.4877, *P* = 0.007), AN (r² = 0.4361, *P* = 0.012), and AP (r² = 0.3989, *P* = 0.018) were the principal soil physicochemical factors influencing fungal community distribution.

**Fig 9:**
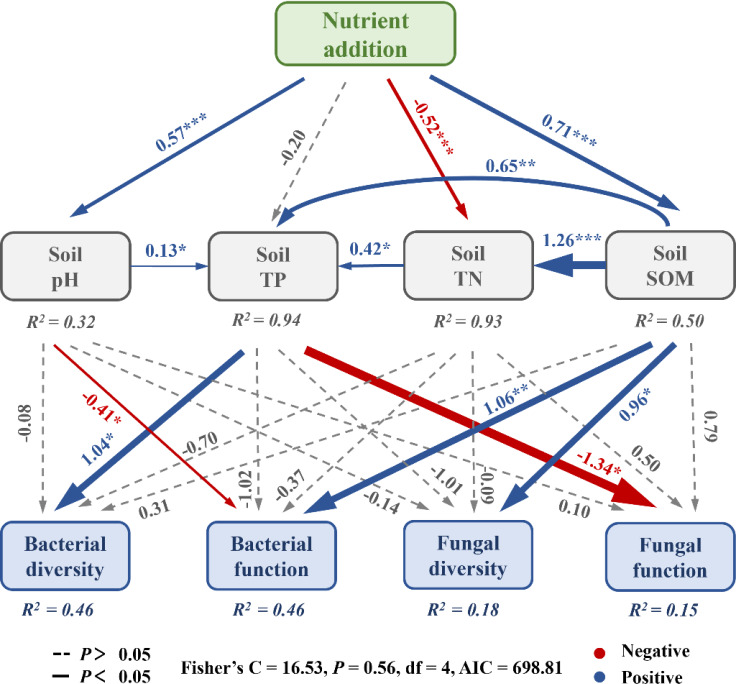
Structural equation modeling analysis of nutrient additions regulating microbial diversity and function by altering soil physicochemical properties. Blue and red arrows signify significant positive and negative impacts, respectively. The numerical values attached to the arrows represent standardized path coefficients. Numbers adjacent to arrows are standardized path coefficients, and the width of the arrow is proportional to the strength of the path coefficient. R^2^ is the proportion of variance explained by the model. Significance levels: * indicates *P* < 0.05, ** indicates *P* < 0.01, and *** indicates *P* < 0.001.

The structural equation model demonstrated Fisher’s C = 16.53, *P* = 0.56, and Akaike information criterion = 698.81, suggesting that the model effectively described the relationships between nutrient addition and soil physicochemical properties, microbial diversity, and functions (Fig. 9). Nutrient addition was found to directly regulate soil pH and SOM, yielding significant positive effects (*P* < 0.001). Further significant positive effects on TN and TP were observed through the indirect regulation of SOM by nutrient addition. TP was the primary factor regulating soil bacterial diversity and fungal functions (*P* < 0.05). Bacterial functions were largely governed by pH and SOM. Additionally, SOM was identified as the primary regulator of soil fungal diversity (*P* < 0.05).

## DISCUSSION

### Effects of nutrient addition on soil physicochemical properties

Artificial intervention strategies aimed at enhancing soil nutrient levels serve as a crucial approach for soil restoration in alpine mining regions (45). This investigation revealed that the application of sheep manure and commercial organic fertilizer, whether used independently or in combination, notably influenced the soil physicochemical properties of alpine mining areas, generally resulting in an increase in soil nutrient content (Table 2, *P* < 0.05). These findings underscore the severely depleted state of soil nutrients in these regions, highlighting the pressing need for external nutrient inputs. Specifically, the combined use of sheep manure and commercial organic fertilizer led to an increase in soil pH, with the M2 treatment demonstrating a markedly higher pH compared to other treatments (Table 2). This effect can be attributed to two primary mechanisms. First, sheep manure and commercial organic fertilizer addition alter the soil’s physical properties, enhancing soil aeration and water retention, which facilitates the accumulation of alkaline substances in the soil (46). Second, both sheep manure and commercial organic fertilizer are rich in SOM; upon application, the increased SOM content stimulates microbial activity in the soil (47). As SOM decomposes, microorganisms release alkaline compounds, such as ammonia, which further contribute to an increase in soil pH (25, 48). Whether through the accumulation or release of alkaline substances, this impact is most evident following the equal-proportion combined application of sheep manure and commercial organic fertilizer, resulting in a markedly elevated pH under the M2 treatment versus the other treatments.

In this study, the content of SOM and available nutrients under the mixed application treatment was found to be markedly higher compared to other treatments. Overall, the M1 treatment led to a notable increase in the concentrations of TN, TP, SOM, AN, and AP in the soil when compared to other treatments. The nutrient components in sheep manure and commercial organic fertilizer likely exhibit complementary effects (49). Sheep manure is typically rich in SOM, nitrogen, phosphorus, and other essential nutrients, whereas commercial organic fertilizer tends to contain higher levels of trace elements and microorganisms (45). The combined application of both fertilizers enables the mutual supplementation of their respective nutrients, thereby enhancing the overall nutritional profile of the soil. Commercial organic fertilizer, being particularly rich in microorganisms, may facilitate the decomposition of SOM in sheep manure, thus accelerating the release of nutrients (50). During this decomposition process, microorganisms are also capable of fixing nitrogen from the atmosphere, which contributes to increased nitrogen levels in the soil (51). The synergistic use of both organic fertilizers not only improves soil physical properties such as aeration, water retention, and permeability but also helps in retaining soil nutrients and enhancing plant nutrient uptake (52). The microorganisms and OM present in commercial organic fertilizer can promote the conversion of fixed phosphorus in the soil into AP, making it more readily available for plant absorption, thus increasing the AP content in the soil (53). When either sheep manure or commercial organic fertilizer is applied individually, nutrients are prone to loss due to soil adsorption and leaching effects (54). However, the combined application of these organic fertilizers helps regulate soil pH, bringing it within a more favorable range for plant growth, thereby improving the effectiveness of soil nutrients (55). In alpine mining areas, where soil substrates are severely deficient, sheep manure provides a relatively higher amount of soil substrate (45). In the M1 treatment, the proportion of sheep manure exceeds that of commercial organic fertilizer. The combined application not only addresses the soil substrate deficiency in alpine mining areas but also markedly enhances soil nutrient content, providing valuable insights for restoring soil quality in such regions.

### Effects of nutrient addition on soil microbial communities

During the soil restoration processes in alpine mining areas, soil microorganisms serve a pivotal function in facilitating ecological restoration and promoting sustainable development by enhancing the physical, chemical, and biological properties of the soil, as well as contributing to ecosystem recovery and stability (25). The present study demonstrated that the application of sheep manure, both alone and in combination with commercial organic fertilizer, markedly influenced soil microbial community diversity (Fig. 1, *P* < 0.05). In contrast, the application of commercial organic fertilizer alone did not lead to substantial changes (Fig. 1, *P* < 0.05). The impact of commercial organic fertilizer on soil microbial diversity is a multifaceted process, shaped by various factors (56). Despite the fact that commercial organic fertilizers contain considerable amounts of SOM, they may exhibit lower diversity when compared to natural organic materials such as animal and plant excreta (47, 57). The diversity of soil microorganisms is governed by both the type and quality of the SOM. Consequently, if commercial organic fertilizers lack specific organic compounds, their ability to markedly alter microbial diversity may be limited (58). Conversely, the combined use of sheep manure and commercial organic fertilizer introduces a broader range of organic compounds, which markedly alters soil microbial diversity (59).

This investigation demonstrated that the application of sheep manure, commercial organic fertilizer, and their combination markedly altered the microbial community structure in soils of alpine mining areas. Such alterations occurred as a result of the introduction of new microbial populations, changes in soil nutritional and physical properties, and modifications to the interactions among microorganisms, all of which contributed to the observed shifts in the microbial community structure (60). Sheep manure, rich in beneficial bacteria, fungi, and protozoa, introduces new microbial species upon its application to soil, thereby enhancing microbial diversity (61). Furthermore, the OM in sheep manure serves as an energy and carbon source for microorganisms, stimulating their growth and reproduction (62). During the composting of commercial organic fertilizer, OM is decomposed by microorganisms, generating considerable heat, which leads to the dominance of certain microbial species. When applied to the soil, these microorganisms are introduced, thus altering the existing microbial community structure (50). The various treatments resulted in significant changes in the relative abundance of dominant soil microorganisms (Fig. 3). For instance, Gemmatimonadota and Mortierellomycota exhibited higher relative abundances under the M1 treatment, while the relative abundance of *Preussia* was markedly lower compared to other treatments. Gemmatimonadota may play a role in maintaining soil fertility and decomposing SOM, contributing to nutrient cycling within the soil (25). Mortierellomycota, key decomposers in soil, are capable of breaking down SOM, thereby facilitating nutrient cycling (63). Moreover, Mortierellomycota interacts with other microorganisms, helping maintain the diversity of soil microbial communities (64). These findings suggest that a healthy soil microbial environment can be achieved through the formulation of a rational mixed application ratio of sheep manure and commercial organic fertilizer. In this study, the M1 treatment not only enhanced soil nutrient content but also improved the microbial environment, making it a potentially effective strategy for promoting soil restoration in alpine mining areas.

The application of sheep board manure and commercial organic fertilizer significantly increased the content of dung rot bacteria in the soil in terms of the overall effect. This is because both manure and commercial organic fertilizers are rich in organic matter, which can provide sufficient carbon, nitrogen, and other nutrients for dung rotting bacteria (65). Sheep plate manure also contains a variety of minerals and trace elements, which can meet the needs of the growth and reproduction of fecal rot bacteria (65). Commercial organic fertilizer, after processing, has a more balanced nutrient composition, but it is also easier to be absorbed and utilized by fecal rot bacteria, which can promote their massive reproduction and increase the content of fecal rot bacteria in the soil. It was also found in this study that sheep slat manure and the combined application of sheep slat manure and commercial organic fertilizer effectively reduced the content of plant pathogenic bacteria. This was due to the value addition of beneficial microorganisms, which were abundant in sheep plate manure, such as Bacillus and Actinomycetes (61). After these beneficial microorganisms colonize the soil, they compete with plant pathogenic bacteria for living space and nutrients, making it difficult for pathogenic bacteria to gain a foothold in the soil. In addition, the aeration and water retention of the soil are enhanced. Increased soil aeration reduces the anaerobic environment and inhibits the growth of some anaerobic pathogenic bacteria. Therefore, it is likely that the application of sheep manure, commercial organic fertilizer, and their combined use promoted changes in soil microbial functions, primarily by providing essential nutrients, improving the soil environment, and enhancing microbial diversity (66).

### Relationships between soil physical and chemical properties and microbial communities

In this study, no significant effects of soil pH on bacterial and fungal diversity were observed (Fig. 7, *P* > 0.05). This could be attributed to the restrictions imposed on the physiological and metabolic processes of soil microorganisms by the extreme environmental conditions in alpine regions, leading to a relatively limited impact of pH on microbial diversity (67). On the other hand, soil chemical properties, such as TN, TP, SOM, AP, and AN, were found to exhibit significant positive correlations with bacterial diversity (Fig. 7a, *P* < 0.05). Nutrients like TN and TP provide essential material foundations that support bacterial growth and reproduction (68). The availability of higher nutrient levels facilitates the growth of a greater variety of bacterial species, thereby increasing bacterial diversity (69). SOM, serving as a primary food source for microorganisms, supports the maintenance of more diverse microbial communities in soils with higher SOM content (70). Additionally, AP and AN, being readily accessible nutrients in the soil, directly influence bacterial growth rates and diversity (71). Elevated concentrations of these available nutrients promote bacterial growth and metabolic activities, which in turn contribute to an increase in bacterial species diversity and abundance (72). Furthermore, soil fungal diversity was found to be positively correlated with TP content (Fig. 7b, *P* < 0.05). Phosphorus is an essential nutrient for fungal growth, and an increase in TP content in the soil can directly stimulate fungal growth and reproduction, thereby enhancing fungal diversity (73). Moreover, a sufficient supply of phosphorus contributes to improved soil environmental stability, which further aids in the maintenance and enhancement of fungal diversity (74, 75).

This study identified TP, SOM, TN, AP, and AN as the primary physicochemical factors influencing the distribution of bacterial communities in soil. TP, AN, and AP were found to be the key physicochemical determinants of fungal community distribution, aligning with the findings of Yu et al. (45). In alpine mining areas, low temperatures and the presence of slag soil were found to restrict nutrient accumulation, suggesting that soil physicochemical properties are likely the primary factors determining the distribution and diversity of bacterial and fungal communities (76). Structural equation modeling analysis indicated that the diversity of soil bacteria and the functionality of fungi were predominantly regulated by TP (Fig. 9, *P* < 0.05). In environments deficient in phosphorus, bacterial growth was limited, leading to reduced bacterial diversity (77). However, the addition or increase of phosphorus in the soil may prompt the succession of bacterial communities, with new microbial species replacing those adapted to low-phosphorus conditions, thus altering bacterial diversity (78). The availability of phosphorus is closely associated with fungal biomass; when TP levels are low, fungal biomass may also be constrained (73). Phosphorus serves a pivotal function in enzyme activity, being a component of various enzymes, and thus TP levels influence fungal enzyme activity, which affects their metabolic processes and ecological functions (74). Bacterial functions were mainly influenced by pH and SOM, due in part to the fact that bacterial metabolic activities are largely reliant on enzymatic catalysis, which is directly affected by soil pH (79). Furthermore, SOM serves as the primary energy and nutrient source for soil bacteria (80). Bacteria acquire carbon and energy by decomposing organic compounds present in SOM (81). As such, the content of SOM directly influences bacterial metabolic functions. Soil fungal diversity was chiefly regulated by SOM (Fig. 9, *P* < 0.05). This is due to the fact that SOM functions as the principal nutritional source for soil fungi (70), containing abundant nutrients like carbon, nitrogen, and phosphorus, which are vital for fungal growth and reproduction (70). When SOM content is high, fungi can access sufficient nutrients, thus supporting greater diversity and activity (82). Overall, the introduction of sheep manure and commercial organic fertilizer in alpine mining areas facilitated changes in microbial structure and function by improving soil physicochemical properties.

### Conclusions

The combined application of sheep manure and commercial organic fertilizer significantly enhanced soil fertility and microbial community structure in the alpine mining region. Among the treatments, M1 (60% sheep manure + 40% commercial organic fertilizer) exhibited the most pronounced improvement, substantially increasing soil organic matter, total nitrogen, total phosphorus, available nitrogen, and available phosphorus. Various nutrient addition treatments led to significant changes in both the structure of the soil microbial community and the abundance of functional microorganisms. The F treatment did not produce significant effects on the OTUs index, Chao index, or Shannon index of the bacterial community in the soil. Similarly, no significant alterations were observed in the Shannon or Simpson indices of the fungal community under different nutrient addition treatments. RDA revealed that TP, SOM, TN, AP, and AN were the primary physicochemical factors influencing the distribution of the bacterial community, while TP, AN, and AP were identified as the critical factors affecting the fungal community distribution. Structural equation modeling analysis suggested that TP was the main regulator of soil bacterial diversity and fungal function. The bacterial function was primarily controlled by pH and SOM, and SOM predominantly influenced soil fungal diversity. In conclusion, the M1 treatment is the most effective strategy for improving soil quality and optimizing microbial communities, providing an optimal fertilization approach for soil restoration in alpine mining areas.

## Data Availability

The raw sequence data reported in this paper have been deposited in the Genome Sequence Archive (Genomics, Proteomics, and Bioinformatics 2021) in National Genomics Data Center (Nucleic Acids Res 2022), China National Center for Bioinformation/Beijing Institute of Genomics, Chinese Academy of Sciences (GSA: CRA021516 and CRA021518) that are publicly accessible at https://ngdc.cncb.ac.cn/gsa.
